# NEAT1 modulates herpes simplex virus-1 replication by regulating viral gene transcription

**DOI:** 10.1007/s00018-016-2398-4

**Published:** 2016-10-25

**Authors:** Ziqiang Wang, Ping Fan, Yiwan Zhao, Shikuan Zhang, Jinhua Lu, Weidong Xie, Yuyang Jiang, Fan Lei, Naihan Xu, Yaou Zhang

**Affiliations:** 1 School of Life Sciences, Tsinghua University, Beijing, 100084 People’s Republic of China; 2 Key Lab in Healthy Science and Technology, Division of Life Science, Graduate School at Shenzhen, Tsinghua University, Shenzhen, 518055 People’s Republic of China; 3 Shenzhen South China Pharmaceutical Co., Ltd, Shenzhen, 518055 People’s Republic of China; 4 The State Key Laboratory Breeding Base-Shenzhen Key Laboratory of Chemical Biology, the Graduate School at Shenzhen, Tsinghua University, Shenzhen, 518055 People’s Republic of China; 5 School of Pharmaceutical Sciences, Tsinghua University, Beijing, 100084 People’s Republic of China; 6 Open FIESTA Center, Tsinghua University, Shenzhen, 518055 People’s Republic of China

**Keywords:** NEAT1, Paraspeckle, HSV-1, STAT3, Viral replication, Gene regulation

## Abstract

Nuclear paraspeckle assembly transcript 1 (NEAT1) is the crucial structural platform of paraspeckles, which is one type of nuclear bodies. As a stress-induced lncRNA, the expression of NEAT1 increases in response to viral infection, but little is known about the role of NEAT1 or paraspeckles in the replication of herpes simplex virus-1 (HSV-1). Here, we demonstrate that HSV-1 infection increases NEAT1 expression and paraspeckle formation in a STAT3-dependent manner. NEAT1 and other paraspeckle protein components, P54nrb and PSPC1, can associate with HSV-1 genomic DNA. By binding with STAT3, PSPC1 is required for the recruitment of STAT3 to paraspeckles and facilitates the interaction between STAT3 and viral gene promoters, finally increasing viral gene expression and viral replication. Furthermore, thermosensitive gel containing NEAT1 siRNA or STAT3 siRNA effectively healed the skin lesions caused by HSV-1 infection in mice. Our results provide insight into the roles of lncRNAs in the epigenetic control of viral genes and into the function of paraspeckles.

**Electronic supplementary material:**

The online version of this article (doi:10.1007/s00018-016-2398-4) contains supplementary material, which is available to authorized users.

## Introduction

NEAT1, a long noncoding RNA (lncRNA) in animal cells, exists in two isoforms: NEAT1v1 (human 3.7 kb; mouse 3.2 kb) and NEAT1v2 (human 22.7 kb; mouse 20 kb). Previous studies have reported the cellular functions of NEAT1, including its function in the retention of inverted Alu repeat-containing RNAs in paraspeckle-associated complexes [1] and its regulation of gene expression by binding to active chromatin sites [2]. NEAT1 has also been shown to provide a crucial structural framework for organizing paraspeckles, which are recently discovered nuclear bodies in mammalian cells with unclear functions [3, 4].

Paraspeckles are found in the nuclei of mammalian cells and are formed by lncRNA and numerous RNA-binding proteins [5]. NEAT1 is an essential architectural component of paraspeckles, and knockdown of NEAT1 results in the disintegration of the paraspeckles [4, 6, 7]. In addition to NEAT1, paraspeckles also contain core protein components, including PSPC1 (paraspeckle component 1), NONO (non-POU domain-containing octamer-binding, p54), SFPQ (splicing factor, proline- and glutamine-rich), CPSF6, and RNA-binding motif 14 (RBM14) [5], and dozens of new paraspeckle proteins have recently been identified [8–10]. Although some functions of the paraspeckle components have been examined, few reports have focused on the functions of paraspeckles as nuclear bodies. To date, the potential functions of paraspeckles include the transcriptional regulation of gene expression by retaining mRNAs in the nucleus [11] and the sequestration of transcription factors from the promoters of target genes [12, 13].

HSV-1 is a human alpha herpesvirus associated with orofacial and genital herpes infections and has been implicated in herpes viral encephalitis [14]. HSV-1 contains a double-stranded DNA and will establish a lifelong latent infection in human trigeminal ganglia following the primary infection. After reactivation, the virus is transmitted through acute infection or asymptomatic shedding, thus causing the extensive spreading of viruses. Upon entry into the host cell, the virus exploits a series of cellular protein factors to facilitate the progression of its life cycle. Host cell factor 1, a transcriptional co-activator, plays a central role in initiating the expression of the immediate early (IE) viral genes by interacting with numerous transcription factors in the host cell, including virion protein 16 [15] and ASF1b, the latter of which mediates the progression of cellular DNA replication forks [16]. HSV-1 also uses early growth response protein 1 to promote IE gene expression when it binds to the key regulatory elements adjacent to HSV-1 IE genes [17]. More host factors have recently been shown to modulate HSV-1 replication by interacting with viral proteins. P32, a target of the viral protein ICP34.5, has been shown to facilitate viral nuclear egress [18], and the DNA methyltransferase DNMT3A was reported to promote HSV-1 replication by associating with VP26, a viral capsid protein [19]. Being the framework of paraspeckles, NEAT1 is a virus-induced lncRNA. To date, NEAT1 expression has been shown to be upregulated during infection by Japanese encephalitis virus [20], rabies virus [20], human immunodeficiency virus type 1 (HIV-1) [24] and HSV-1 [13]. In HIV-1 infection, NEAT1 modulates viral replication through the nuclear retention of some important RNAs that play active roles in viral gene expression [24]. However, the importance of NEAT1 in HSV-1 replication is currently unclear.

Here, we investigated the role of NEAT1 and paraspeckles in HSV-1 replication and viral gene expression. We found that HSV-1 infection upregulates NEAT1 expression and paraspeckle formation in a STAT3-dependent manner. NEAT1 and other paraspeckle components can bind to the viral genes and help them retain in paraspeckles. PSPC1, a component of paraspeckles, is required for the recruitment of STAT3 to paraspeckles and facilitates the interaction between viral genes and STAT3, finally increasing viral gene expression and viral replication.

## Results

### HSV-1 infection increases NEAT1 expression and paraspeckle formation in a STAT3-dependent manner

**Fig. 1:**
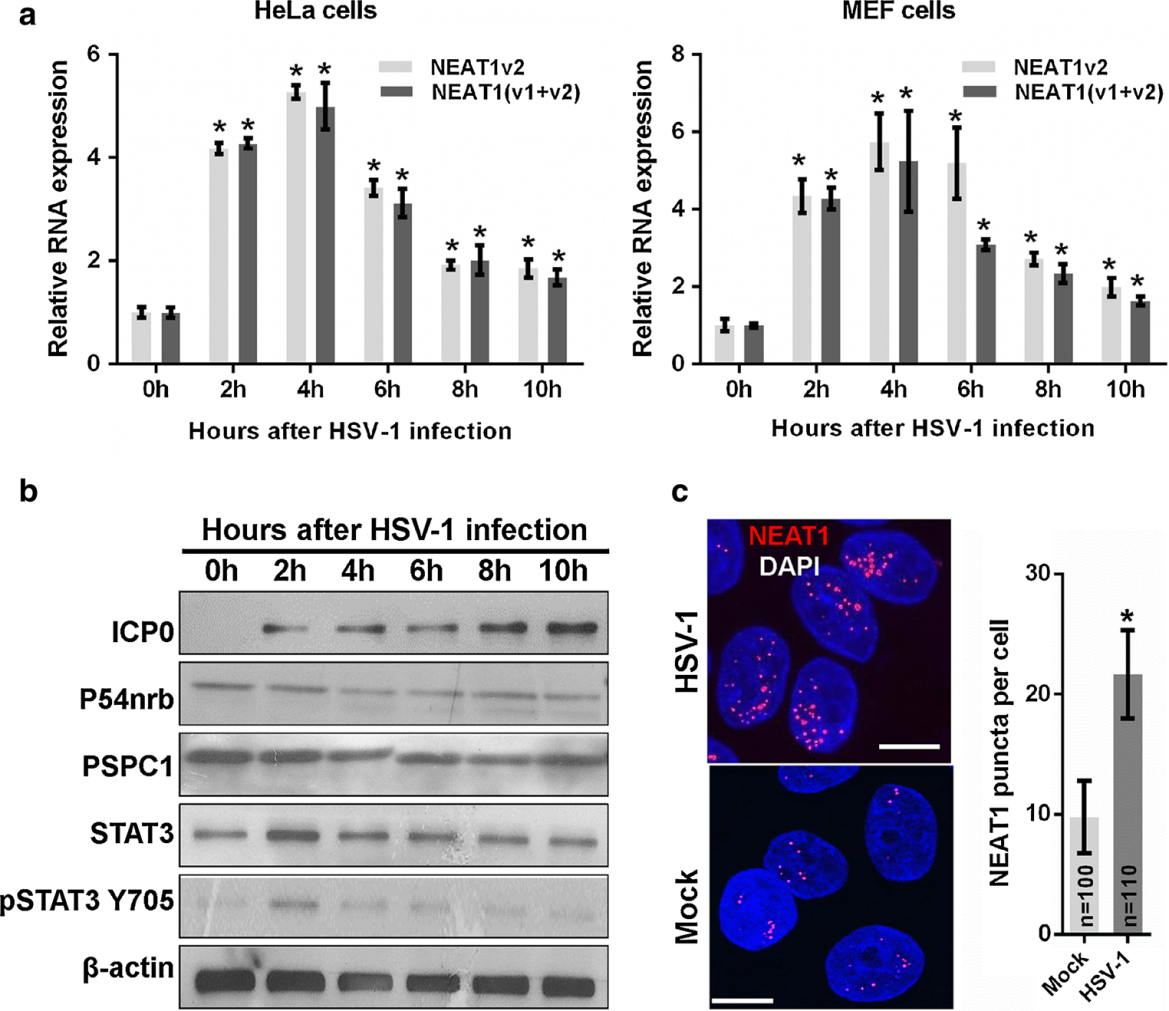
HSV-1 infection increases NEAT1 expression and paraspeckle formation. **a** HeLa and MEF cells were infected with HSV-1 and harvested at the indicated time points. The expression levels of NEAT1 relative to those of β-actin mRNA were determined with real-time PCR. The data were normalized to the NEAT1 level at 0 h after HSV-1 infection. **b** HeLa cells were infected with HSV-1 and collected at the indicated time points for western blot to analyze the expression of ICP0, P54nrb, PSPC1, STAT3, pSTAT3 Y705 and β-actin. **c** HeLa cells were fixed 4 h after HSV-1 infection and incubated with the NEAT1 probe (*red*). Fluorescence images were captured with a confocal microscope. *Scale bars* 10 μm. The number of NEAT1 puncta per cell was analyzed. *p < 0.01

It has been reported that NEAT1 is induced in host cells by viral infection, including HSV-1 infection [13]. However, the mechanism underlying this induction remains unclear. In this study, we first investigated the expression patterns of NEAT1 during HSV-1 infection in HeLa cells and mouse embryonic fibroblast (MEF) cells. We found that NEAT1 expression increased 2 h after HSV-1 infection and peaked at 4 h, gradually declining thereafter in both cell types (Fig. 1a). Because NEAT1 is one of the paraspeckle components, we also examined the effects of HSV-1 infection on the expression of other paraspeckle components. Immunoblotting revealed that viral infection does not affect the expression of the paraspeckle protein components P54nrb and PSPC1 in HeLa cells (Fig. 1b). To study the effects of HSV-1 infection on paraspeckle formation, we also designed a human NEAT1 probe that consists of a set of Quasar^®^ 570-labeled oligos and targets the middle segment (3800–11,700 bp) of human NEAT1v2. Using an RNA fluorescence in situ hybridization (FISH) assay, we found that HSV-1 infection increases the number of NEAT1 puncta (Fig. 1c), suggesting that viral infection induces the formation of paraspeckles, possibly by increasing the expression of NEAT1, which is the organizer of paraspeckles.

**Fig. 2:**
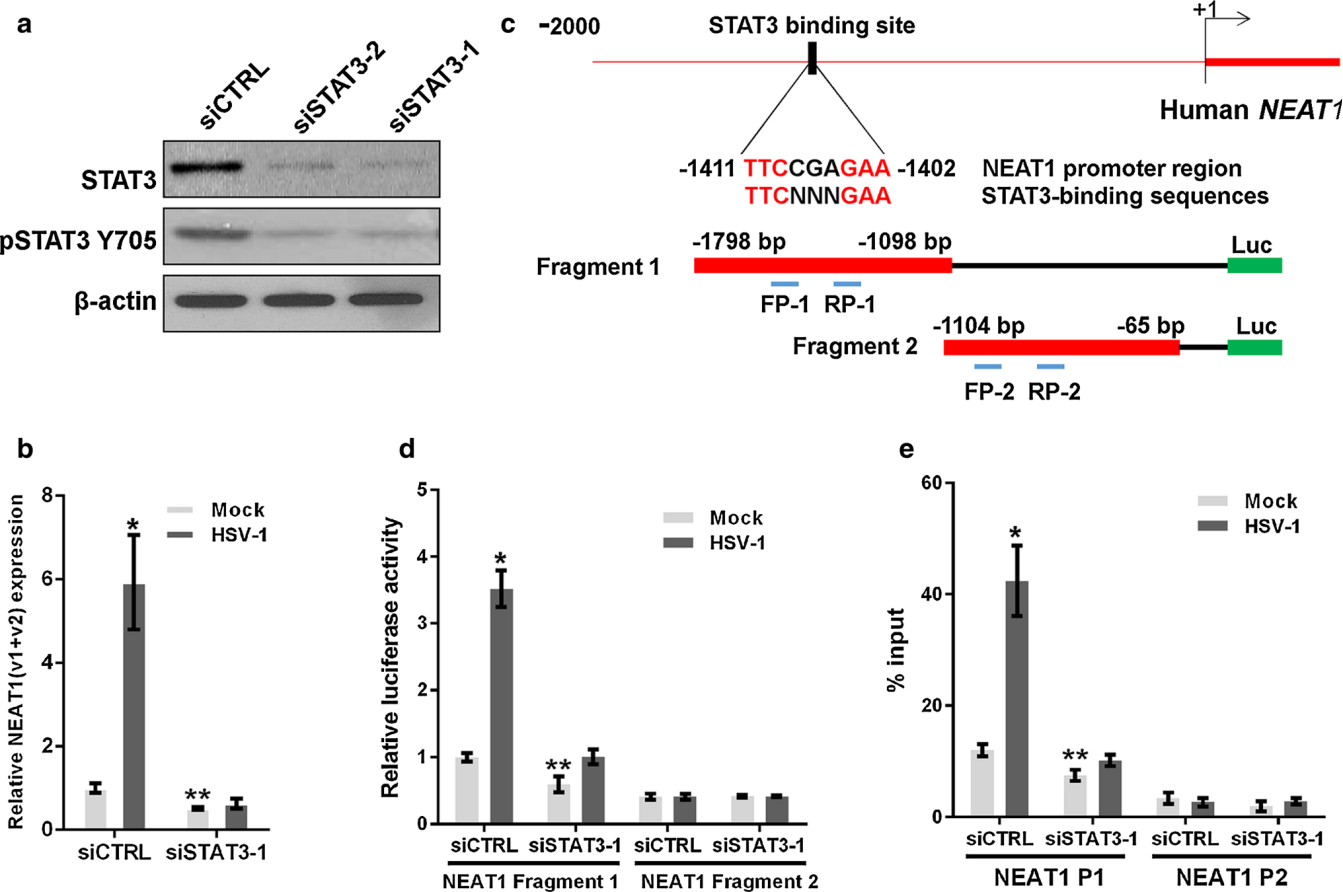
STAT3 is involved in NEAT1-expression-mediated HSV-1 infection. **a** HeLa cells were transfected with STAT3 siRNA or the negative control siRNA for 36 h. The levels of STAT3 and pSTAT3 Y705 were determined by western blotting. **b** HeLa cells transfected with STAT3 or the control siRNA were mock infected or infected with HSV-1 for 4 h. Relative NEAT1(v1 + v2) levels (compared with β-actin mRNA) were analyzed with real-time PCR. The data were normalized to the control level in mock-infected cells. **c** Schematic representation of the STAT3-binding site in the human *NEAT1* gene. The *black box* shows the potential binding site, and the *red* characters indicate matching sequences. Two luciferase promoter reporter constructs are shown, designated Fragment 1 and Fragment 2. FP: forward primer; *RP* reverse primer. **d** Luciferase activity assay in HeLa cells transfected with STAT3 siRNA and the luciferase reporter containing either Fragment 1 or Fragment 2 of the *NEAT1* gene. The data were normalized to the control level in mock-infected cells. **e** ChIP assays were performed with an anti-pSTAT3 Y705 antibody to determine the fold enrichment of the NEAT1 fragment by pSTAT3 Y705. NEAT1 P1 and NEAT1 P2 refer to the region that was amplified by paired primers (FP1/RP1 and FP2/RP2, respectively). NEAT1 P2 was used as a negative control. *p < 0.01, **p < 0.05

It has been reported that STAT3 is rapidly activated in human amniotic FL cells and leukemic CCRF-CEM cells following HSV-1 infection [21]. We demonstrated that the expression of STAT3 and the level of phosphorylated STAT3 Y705 (pSTAT3 Y705) increased in HeLa cells 2 h after HSV-1 infection and then returned to the basal level (Fig. 1b). Therefore, we investigated whether the increase in NEAT1 expression induced by viral infection is mediated by STAT3. To clarify the relationship between NEAT1 and STAT3, we silenced STAT3 expression with STAT3 siRNA (siSTAT3) (Fig. 2a) and found that silencing STAT3 decreased NEAT1 expression induced by HSV-1 infection (Fig. 2b).

Because STAT3 is an important transcription factor, we thus hypothesized that STAT3 directly regulates the transcriptional activity of the *NEAT1* gene. As expected, we identified a general consensus STAT3-binding motif [22, 23] located between 1411 and 1402 bp upstream of the human *NEAT1* transcription start site (TSS) (Fig. 2c). To investigate the transcription of the *NEAT1* gene, we generated two luciferase promoter reporter constructs by inserting the fragments with or without the STAT3-binding motif, named fragment 1 or fragment 2, into the reporter construct (Fig. 2c). As shown in Fig. 2d, HSV-1 infection enhanced the luciferase activity of *NEAT1* only in the cells co-transfected with the construct containing the STAT3-binding motif and the control siRNA, whereas STAT3 siRNA dramatically decreased luciferase activity, indicating that STAT3 is required for the transcription of *NEAT1* induced by HSV-1 infection. Additionally, we purchased two luciferase reporter constructs that inserted the −1561 and −1251 bp upstream sequences from *NEAT1* TSS, one of which contained the STAT3-binding motif (NEAT1 WT), and one that contained a deletion in the STAT3-binding motif (NEAT1 MUT). The luciferase assay demonstrated that, in contrast to the NEAT1 WT construct, neither HSV-1 infection nor STAT3 siRNA could alter the luciferase activity of the NEAT1 MUT construct (Fig. S1). To confirm that STAT3 interacts with the *NEAT1* promoter, a chromatin immunoprecipitation (ChIP) assay was performed. The results demonstrated that the depletion of STAT3 significantly reduced the fold enrichment of the *NEAT1* P1 fragment containing the STAT3-binding motif in both HSV-1-infected and mock-infected cells, suggesting that STAT3 regulates *NEAT1* expression by interacting directly with the upstream sequence of *NEAT1* (Fig. 2e).

### NEAT1 modulates HSV-1 replication and viral gene expression

**Fig. 3:**
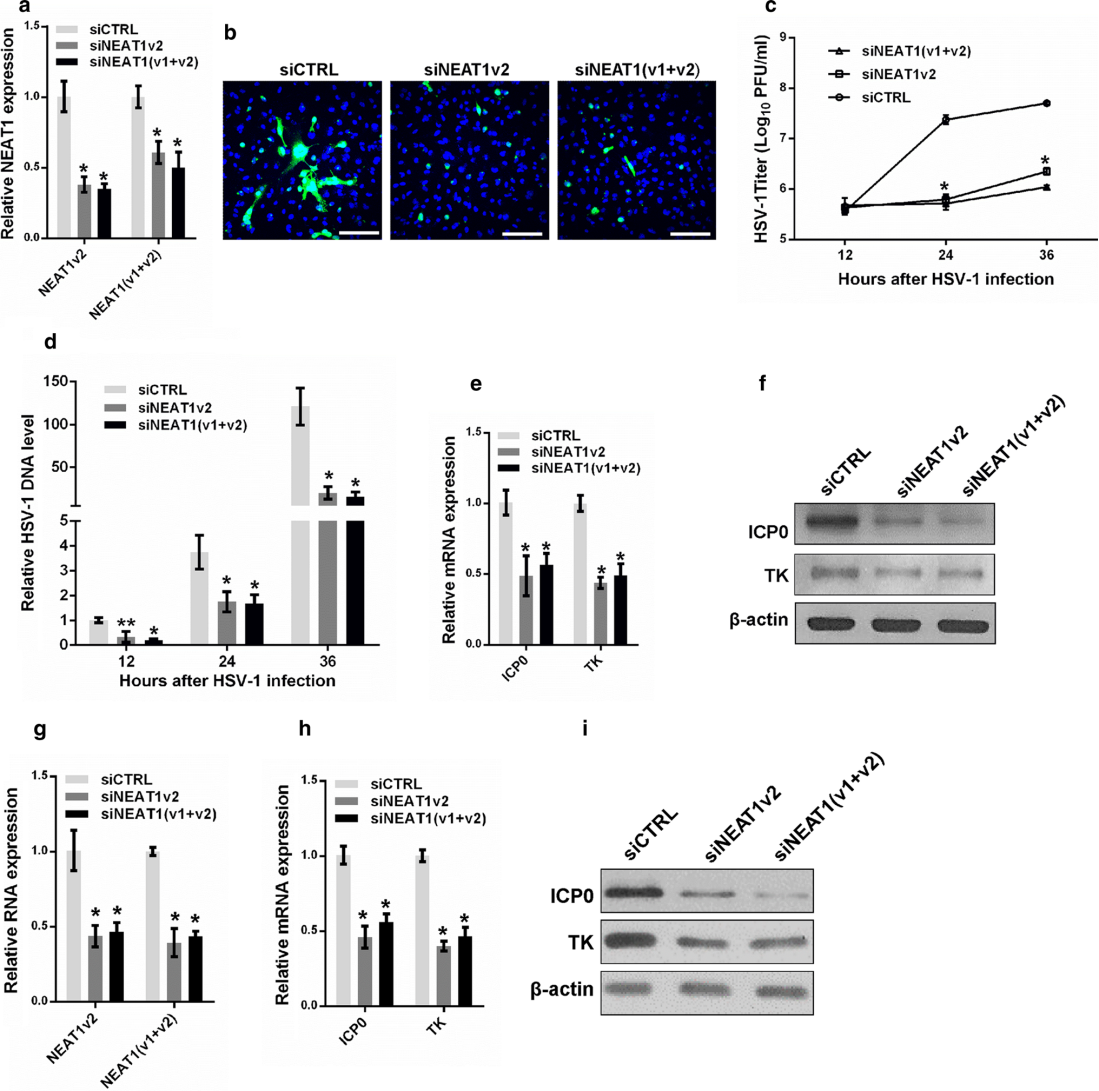
NEAT1 modulates HSV-1 replication and viral gene expression. **a** Analysis of NEAT1 expression in HeLa cells transfected with NEAT1 siRNA or the negative control for 36 h. **b** HeLa cells transfected with NEAT siRNA were infected with HSV-1 and immuno-stained with an anti-HSV-1 glycoprotein antibody (*green*). Images were captured with a confocal microscope. Nuclei were stained with DAPI (*blue*). *Scale bars* 80 μm. **c** Plaque-forming assay in HeLa cells transfected with NEAT siRNA and infected with HSV-1 at the indicated time points. **d** Quantification of viral DNA levels in HeLa cells transfected with NEAT1 siRNA and infected with HSV-1 at indicated time points. **e** The relative mRNA expression levels of ICP0 and TK relative to that of *ACTB* were determined in HeLa cells transfected with NEAT1 siRNA and infected with HSV-1 for 4 h. **f** The samples in **e** were subjected to western blot to analyze the expression of ICP0, TK and β-actin. **g** Analysis of NEAT1 expression in MEF cells transfected with NEAT1 siRNA or the negative control for 36 h. **h** The relative mRNA expression levels of ICP0 and TK were determined in MEF cells transfected with NEAT1 siRNA and infected with HSV-1 for 4 h. **i** Western blot analysis of ICP0, TK and β-actin in MEF cells transfected with NEAT1 siRNA and infected with HSV-1 for 4 h. *p < 0.01, **p < 0.05

To clarify the roles of NEAT1 in HSV-1 replication, we transfected NEAT1-targeting siRNA into HeLa cells and infected the cells with HSV-1 before fixing the cells for HSV-1 glycoprotein staining or collecting the cells for viral gene expression assays (Fig. 3a). The HSV-1 glycoprotein density and intensity were much lower in the NEAT1 knockdown HeLa cells than the control cells (Fig. 3b), suggesting that NEAT1 influences HSV-1 replication and spread. Knocking down NEAT1 expression also markedly inhibited plaque formation 24 and 36 h after HSV-1 infection (Fig. 3c), indicating that less mature viruses were produced after the depletion of NEAT1. We also quantified the effects of NEAT1 on the expression of viral DNA. The thymidine kinase gene (TK) was used to measure the levels of viral DNA; the level of this gene was significantly reduced in the NEAT1 knockdown cells after HSV-1 infection (Fig. 3d). Both real-time PCR and immunoblotting revealed that depletion of NEAT1 caused a reduction in ICP0 and TK gene expression in HSV-1-infected HeLa cells (Fig. 3e, f). Similar results were obtained in MEF cells (Fig. 3g–i). These findings indicate that NEAT1 plays a vital role in HSV-1 DNA replication and viral gene expression.

### NEAT1, P54nrb and PSPC1 co-localize with the HSV-1 genome in paraspeckles

**Fig. 4:**
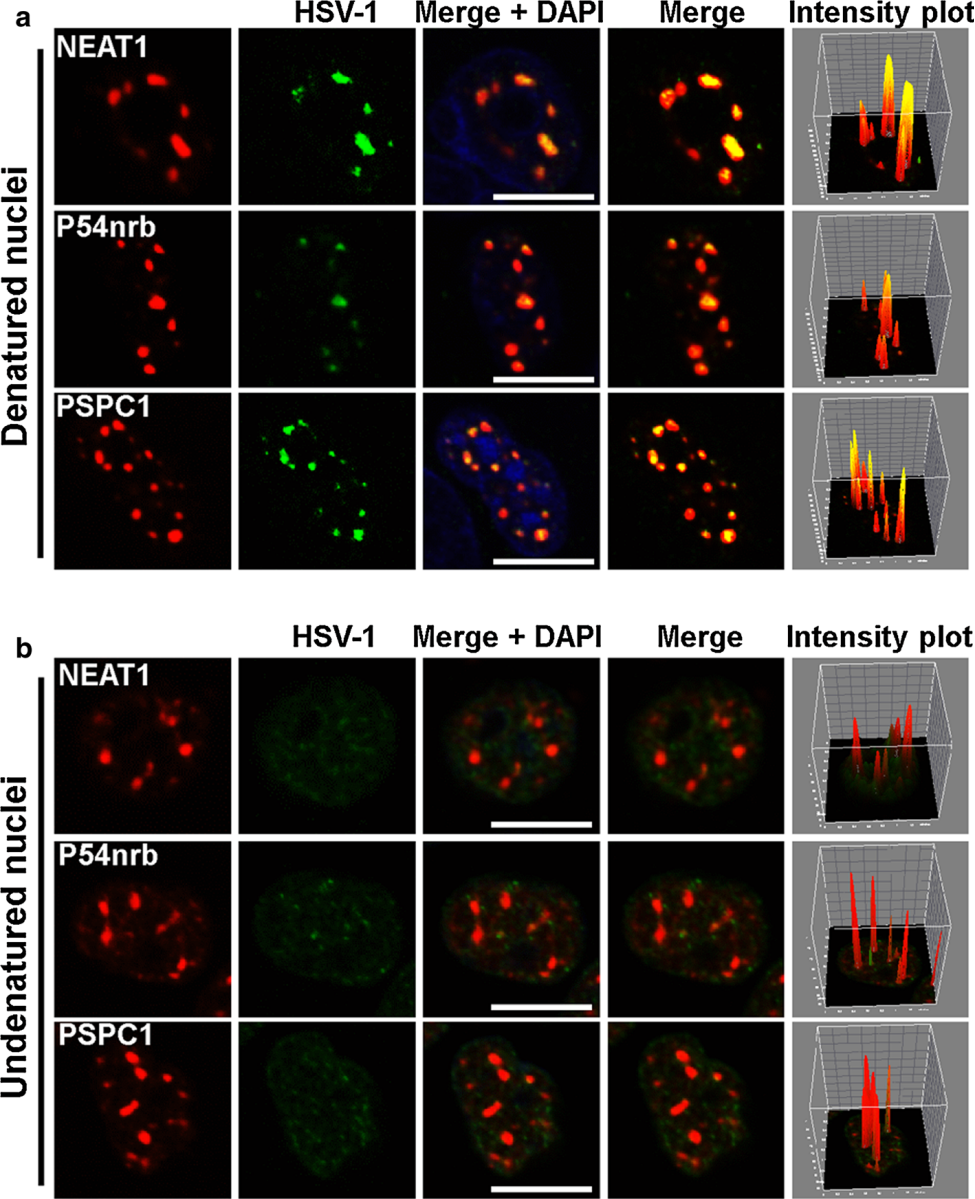
NEAT1, P54nrb, and PSPC1 co-localize with HSV-1 genomic DNA. The biotin-labeled HSV-1 genomic DNA probe (green) incubated with denatured (**a**) or undenatured (**b**) HeLa cells nuclei infected with HSV-1(*blue*) and then incubated with the NEAT1 probe (*red*), the anti-P54nrb antibody (*red*) or anti-PSPC1 antibodies (*red*). The images were captured with a confocal microscope. The intensity plots for the red and green channels were analyzed with ImageJ software. DAPI (*blue*) was used to stain the nuclei. *Scale bars* 10 μm

To understand the mechanism by which NEAT1, an lncRNA, regulates HSV-1 gene expression, we performed an RNA and DNA co-FISH analysis to determine whether NEAT1 physically interacts with the HSV-1 genome. To visualize the HSV-1 genome, we purified and sonicated HSV-1 genomic DNA and labeled it with biotin at its 3′ end. As shown in Fig. 4a, most NEAT1 puncta co-localized with the viral DNA. The paraspeckle components P54nrb and PSPC1 also co-localized with the HSV-1 genome. To determine if the signal generated by biotinylated HSV-1 genomic DNA came from HSV-1 genomic DNA, we performed DNA FISH on undenatured nuclei because DNA would not be detected in undenatured nuclei while RNA would be detected. As shown in Fig. 4b, the signal from biotinylated HSV-1 genomic DNA (green) was dispersed throughout the nucleus and did not co-localize with paraspeckle components (red). These results demonstrate for the first time that paraspeckle components co-localize with HSV-1 DNA, suggesting that the HSV-1 genome is recruited to the nuclear paraspeckle sub-structure to regulate its replication and gene expression.

### NEAT1, P54nrb, and PSPC1 regulate the transcription of viral genes through viral gene promoters

**Fig. 5:**
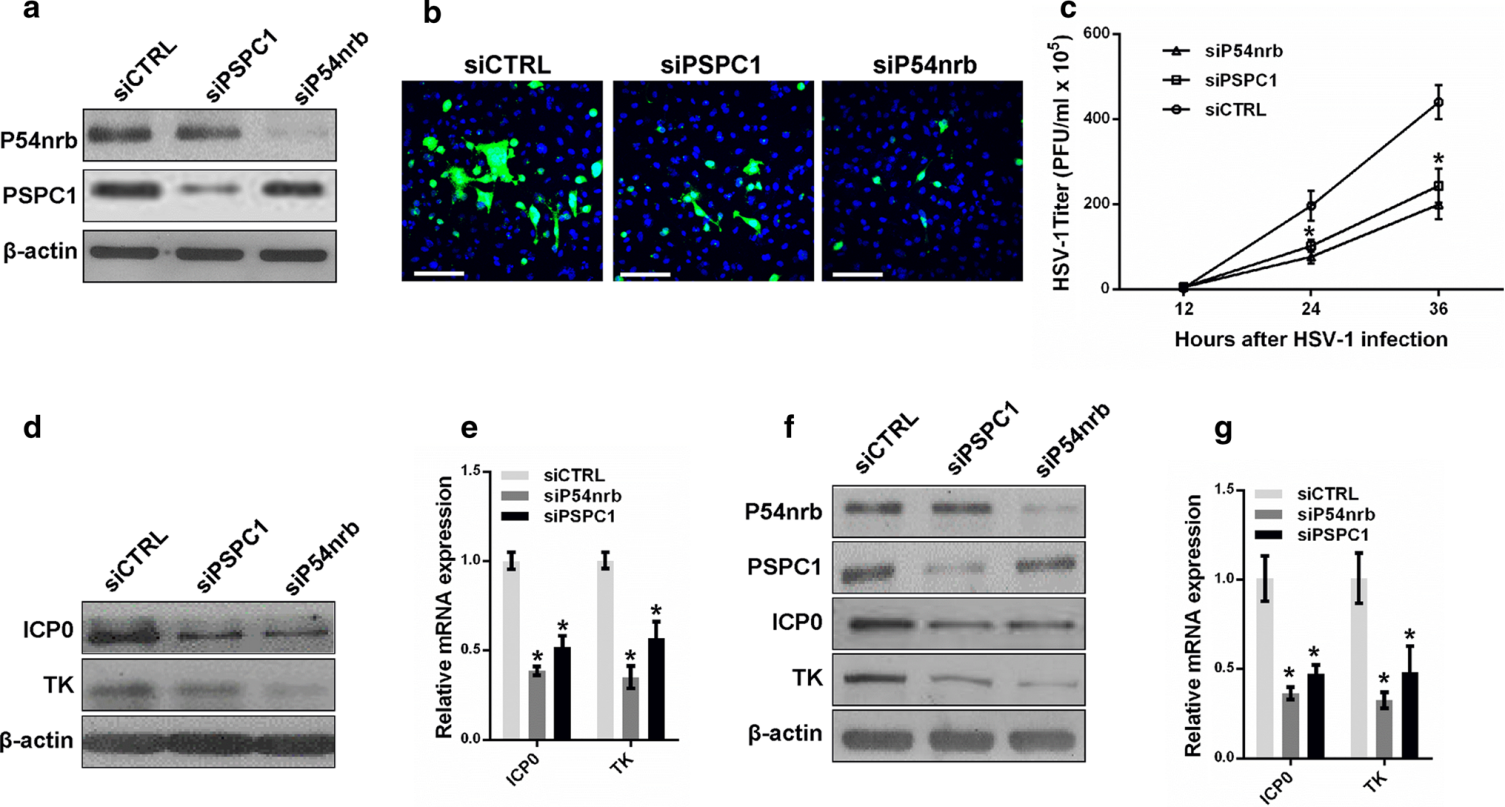
P54nrb and PSPC1 modulate HSV-1 replication and gene expression. **a** HeLa cells transfected with P54nrb, PSPC1 or the control siRNA were infected with HSV-1 for 4 h. The samples were collected for western blot to analyze the expression of P54nrb, PSPC1 and β-actin. **b** The samples in a. were fixed and immuno-stained with an anti-HSV-1 glycoprotein antibody (*green*). Images were captured with a confocal microscope. Nuclei were stained with DAPI (*blue*). *Scale bars* 80 μm. **c** Plaque-forming assay in HeLa cells transfected with P54nrb, PSPC1 or the control siRNA and infected with HSV-1 for the indicated time points. **d** Western blot analysis of ICP0, TK and β-actin in HeLa cells transfected with P54nrb or PSPC1 siRNA and infected with HSV-1. **e** HeLa cells transfected with the indicated siRNAs were infected with HSV-1 for 4 h. The relative expression of ICP0 and TK relative to that of *ACTB* was measured with real-time PCR. **f** MEF cells transfected with the indicated siRNAs were infected with HSV-1 for 4 h. Samples were collected for western blot to analyze the expression of P54nrb, PSPC1, ICP0, and TK. **g** The samples in **f** were collected to analyze the mRNA levels of ICP0 and TK relative to that of *ACTB*. *p < 0.01

Next, we investigated whether the paraspeckle components P54nrb and PSPC1 are also involved in HSV-1 replication. HeLa and MEF cells were transfected with P54nrb or PSPC1 siRNA followed by HSV-1 virus infection (Fig. 5a). An HSV-1 glycoprotein staining assay, a plaque-forming assay, and an HSV-1 gene expression assay were then performed. Depletion of P54nrb or PSPC1 reduced the levels of HSV-1 glycoprotein (Fig. 5b), the viral titer (Fig. 5c), and viral gene expression in both HeLa and MEF cells (Fig. 5d–g).

**Fig. 6:**
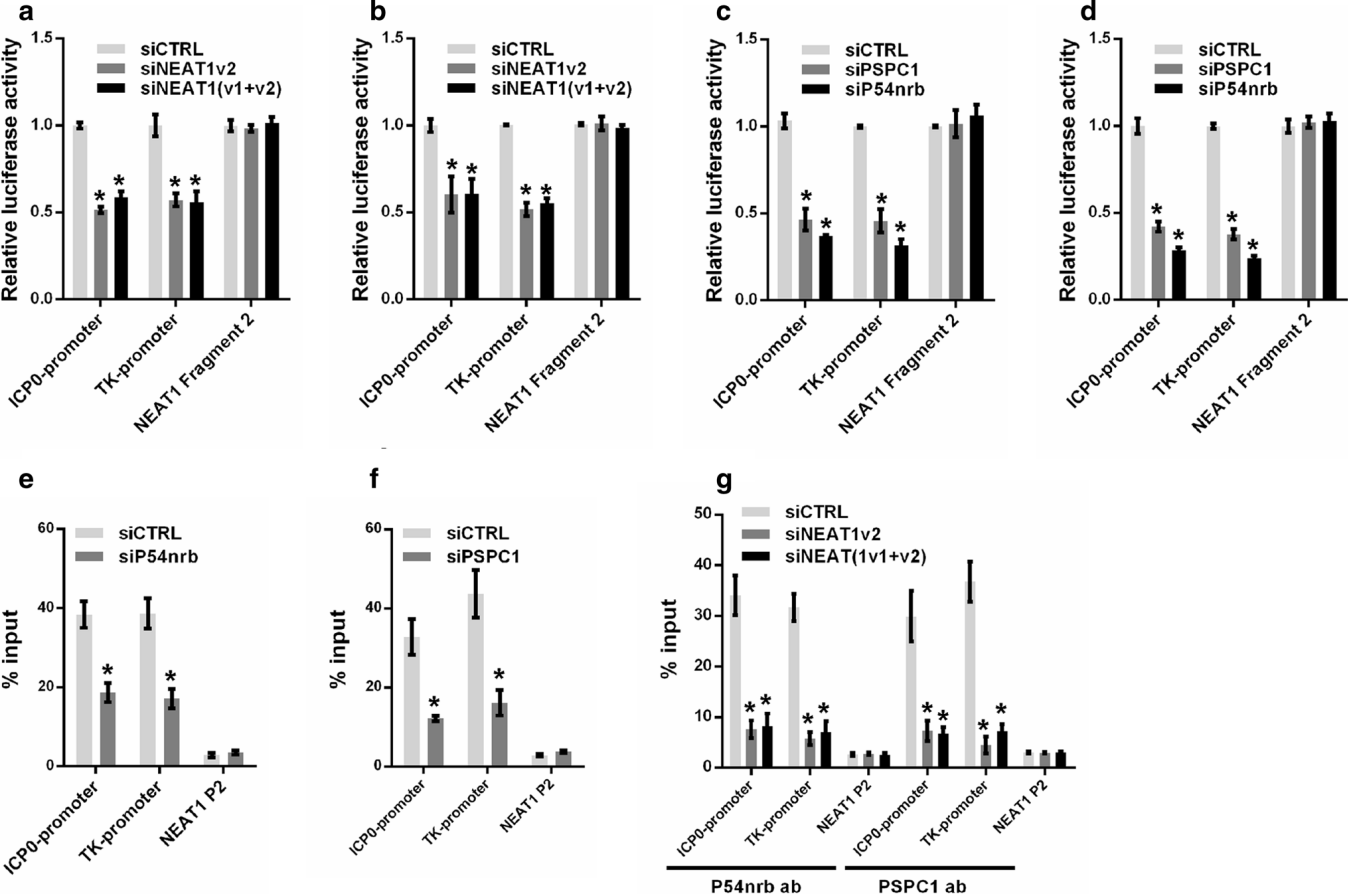
NEAT1 and paraspeckle protein components regulate viral gene transcription. Luciferase activity assay in HeLa (**a**) or MEF cells (**b**) co-transfected with NEAT siRNA and the luciferase reporter plasmid containing either the ICP0 promoter or the TK promoter. NEAT1 Fragment 2 reporter was used as the control. Luciferase activity assay in HeLa (**c**) or MEF cells (**d**) co-transfected with P54nrb or PSPC1 siRNA and a luciferase reporter plasmid containing either the ICP0 promoter or the TK promoter. The NEAT1 Fragment 2 reporter was used as the control. HeLa cells transfected with P54nrb siRNA (**e**) or PSPC1 siRNA (**f**) were infected with HSV-1 for 4 h. A ChIP assay was performed with anti-P54nrb or anti-PSPC1 antibody, and the fold enrichment of the ICP0 or TK promoter by P54nrb or PSPC1 relative to the input level was determined with real-time PCR. NEAT1 P2 refers to the region of NEAT1 Fragment 2 that was amplified by paired primers (FP2 and RP2, in Fig. 2b) and was used as a negative control. **g** HeLa cells transfected with NEAT1 siRNA were infected with HSV-1 for 4 h. ChIP assays were performed with anti-P54nrb or anti-PSPC1 antibody, and the fold enrichment of the ICP0 and TK promoters by P54nrb or PSPC1 relative to the input level was examined with real-time PCR. NEAT1 P2 refers to the region of NEAT1 Fragment 2 that was amplified by paired primers (FP2 and RP2, in Fig. 2b) and was used as a negative control. *p < 0.01

To further understand the role of paraspeckles in regulating HSV-1 gene expression, we constructed a luciferase reporter containing the promoter fragment of either ICP0 or the HSV-1 TK gene. Knockdown of NEAT1 inhibited the transcriptional activities of both the ICP0 promoter and the TK promoter in HeLa and MEF cells (Fig. 6a, b). We also analyzed the effects of P54nrb and PSPC1 knockdown on the transcription of the ICP0 and TK genes using the same assay. As expected, the depletion of P54nrb or PSPC1 reduced the transcription of both ICP0 and TK in HeLa and MEF cells (Fig. 6c, d). A ChIP assay also confirmed that depletion of P54nrb or PSPC1 reduced the number of ICP0 and TK promoters bound to P54nrb or PSPC1 antibodies (Fig. 6e, f).

Because NEAT1 provides the structural scaffold that organizes protein components to form paraspeckles, we investigated whether the binding of P54nrb and PSPC1 to the ICP0 and TK promoters is regulated by NEAT1. As expected, the fold enrichment of the ICP0 and TK promoters associated with P54nrb and PSPC1 was markedly reduced by NEAT1 siRNA (Fig. 6g). Taken together, these results demonstrate that the paraspeckle protein components P54nrb and PSPC1 are also involved in HSV-1 replication and bind to viral genes. NEAT1 may function as a scaffold that facilitates the interaction between the paraspeckle protein components and the viral genes.

### NEAT1 and paraspeckle protein components regulate STAT3-mediated viral gene expression

Because NEAT1 is upregulated by HSV-1 infection in a STAT3-dependent manner, we were also interested to know if STAT3 is directly involved in the NEAT1-mediated regulation of HSV-1 replication. Overexpression of STAT3 greatly increased HSV-1 replication (Fig. S2a, c) and viral gene expression (Fig. S3b, d), whereas STAT3 knockdown dramatically reduced viral replication (Fig. S2b, d) and viral gene expression (Fig. S3a, c).

**Fig. 7:**
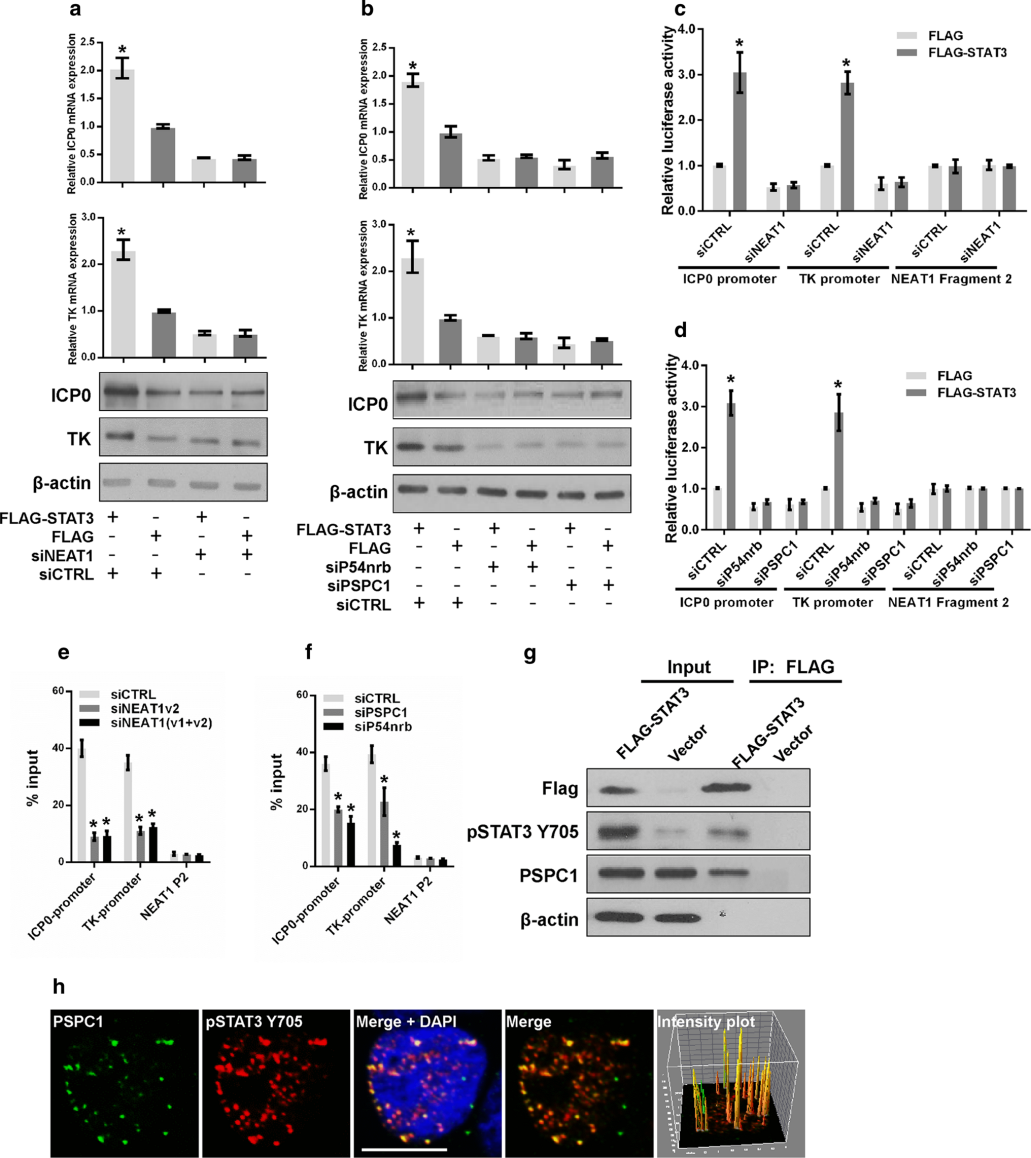
NEAT1 and paraspeckle protein components regulate STAT3-mediated viral gene expression. **a** HeLa cells co-transfected with FLAG-STAT3 and NEAT siRNA were infected with HSV-1. The levels of ICP0 and TK were measured with real-time PCR and western blotting. **b** HeLa cells co-transfected with FLAG–STAT3 and the indicated siRNAs were subjected to HSV-1 infection. The levels of ICP0 and TK were analyzed with real-time PCR and western blotting. HeLa cells were co-transfected with FLAG–STAT3 and NEAT1 siRNA (**c**) or FLAG–STAT3 and P54nrb, PSPC1 siRNAs (**d**) and luciferase reporter plasmids. Cells were infected with HSV-1 for 4 h and collected for the luciferase activity assay. The NEAT1 Fragment 2 reporter was used as the control. HeLa cells infected with NEAT1 siRNA (**e**) or PSPC1 and P54nrb siRNAs (**f**) were subjected to HSV-1 infection. Samples were collected for ChIP assays to analyze the fold enrichment of the ICP0 and TK promoters by pSTAT3 Y705 relative to the input level. NEAT1 P2 refers to the region of NEAT1 Fragment 2 that was amplified by paired primers (FP2 and RP2, in Fig. 2b) and was used as a negative control. **g** HeLa cells transfected with FLAG-STAT3 or the empty vector were infected with HSV-1 for 4 h. The cell lysates were collected and immuno-precipitated with an anti-FLAG antibody. The immuno-precipitates were subjected to western blotting analysis of FLAG, pSTAT3 Y705, PSPC1 and β-actin. **h** HeLa cells infected with HSV-1 were immuno-stained with PSPC1 (*green*) and pSTAT3 Y705 (*red*) and subjected to confocal analysis. The intensity plots for the red and green channels were analyzed with ImageJ software. *Scale bars* 10 μm. *p < 0.01

To determine whether the paraspeckle components are involved in STAT3-mediated viral gene expression, we knocked down NEAT1, P54nrb, or PSPC1 in HeLa cells overexpressing STAT3 or control vectors. Knockdown of NEAT1, P54nrb, or PSPC1 abolished the effect of exogenous STAT3 on viral gene expression (Fig. 7a, b), suggesting that depletion of the paraspeckle components may impair the transcriptional activity of STAT3.

STAT3 is a well-studied transcription factor that regulates gene expression by binding to the promoters of its target genes. We performed a luciferase assay to investigate whether STAT3 regulates the transcription of ICP0 and TK by interacting with their promoters. Knockdown of STAT3 reduced the transcriptional activities of the ICP0 and TK promoters (Fig. S3e), whereas overexpression of STAT3 increased their transcriptional activities (Fig. S3f). To identify the STAT3-binding sites within the ICP0 and TK promoters, we designed primer pairs either upstream or within the coding regions of the ICP0 and TK genes and performed a ChIP assay with an anti-pSTAT3 Y705 antibody in HSV-1-infected HeLa cells (Fig. S4a). pSTAT3 Y705 was enriched at the P2 region (−227 to −65) of the ICP0 gene and the P2 region (−107 to 50) of the TK gene (Fig. S4b), and a general consensus STAT3-binding motif was identified within these regions. STAT3 knockdown significantly reduced the enrichment of pSTAT3 Y705 on the promoters of both the ICP0 and TK genes (Fig. S4c). We further tested the effects of NEAT1, P54nrb, and PSPC1 on STAT3-mediated viral gene transcription. Depletion of NEAT1, P54nrb, or PSPC1 inhibited the transcriptional activities promoted by exogenous STAT3 (Fig. 7c, d). We also performed a ChIP assay to verify whether the binding of STAT3 to the ICP0 and TK promoters was affected by the knockdown of NEAT1, P54nrb, or PSPC1. As expected, the enrichment of the STAT3-associated ICP0 and TK genes declined significantly after the depletion of NEAT1, P54nrb, or PSPC1 (Fig. 7e, f). Moreover, both immunostaining and the immunoprecipitation assay demonstrated that PSPC1 co-localizes with STAT3 (Fig. 7g, h). Furthermore, another immunofluorescence assay was conducted with pSTAT3 Y705 and NEAT1 in HSV-1-infected HeLa cells transfected with PSPC1 siRNA (siPSPC1) or negative control siRNA (siCTRL). The results demonstrated that pSTAT3 Y705 co-localized with NEAT1 and that the inhibition of endogenous PSPC1 could disrupt this association (Fig. S5), indicating that PSPC1 is required for the recruitment of STAT3 to paraspeckles to regulate viral gene expression.

Additionally, we investigated the role of SFPQ in HSV-1 infection. The results demonstrated that SFPQ plays a negative role in viral gene expression in HSV-1-infected HeLa cells (Fig. S6a, b). Moreover, knockdown of SFPQ resulted in the increased transcription of ICP0 and TK (Fig. S6c). To understand the role of SFPQ in STAT3-mediated viral gene expression, we used a ChIP assay to analyze the fold enrichment of the ICP0 and TK promoters by pSTAT3 Y705 in HSV-1-infected HeLa cells transfected with SFPQ siRNA or negative control siRNA. The results demonstrated that knockdown of SFPQ facilitated the interaction between STAT3 and viral gene promoters (Fig. S6d).

### NEAT1 and STAT3 are potential therapeutic targets to limit viral replication

**Fig. 8:**
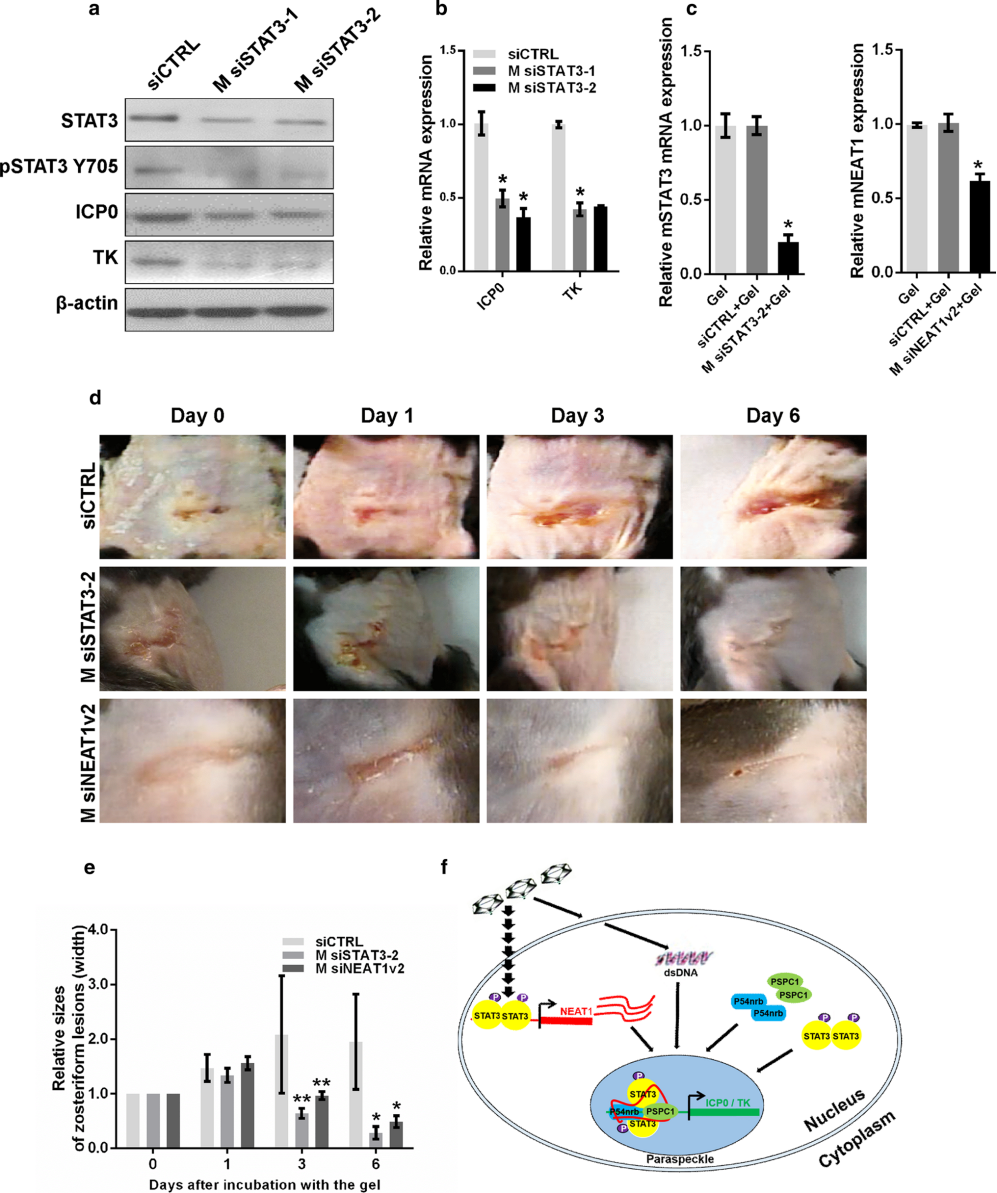
Depletion of STAT3 reduces the development of zosteriform lesions. **a** MEF cells transfected with STAT3 siRNA or negative control siRNA were infected with HSV-1, and the expression levels of STAT3, pSTAT3 Y705, ICP0, and TK were analyzed with western blot. **b** Quantification of the mRNA levels of ICP0 and TK in MEF cells transfected with STAT3 siRNA and infected with HSV-1 with real-time PCR. **c** Thermosensitive gel (100 μL) containing M siSTAT3-2, M siNEAT1v2, negative control, or mock was placed on the skin of C57BL/6 mice. Two days later, the skin was cut off, and the cell lysates were harvested. The relative expression of STAT3 or NEAT1 was determined with real-time PCR. **d** The mice that developed zosteriform lesions were treated with thermosensitive gel containing M siSTAT3-2, M siNEAT1v2 or the negative control siRNA on their skin. The zosteriform lesions were observed on days 0, 1, 3, and 6 after incubation with the gel. The relative sizes of the zosteriform lesions were quantified by measuring the widths of the zosteriform lesions at indicated time points (**e**). **p < 0.05. **f**. Schematic model of the roles of NEAT1, P54nrb and PSPC1 in viral gene expression

Lastly, from a potentially clinical perspective, we sought to determine if knockdown of NEAT1 or STAT3 could limit skin lesions caused by HSV-1 infection in a mouse model. We transfected MEF cells with siRNA targeting *mouse* STAT3 (M siSTAT3-1 and M siSTAT3-2) and then infected the cells with HSV-1. Depletion of STAT3 reduced the expression of ICP0 and TK in HSV-1-infected MEF cells (Fig. 8a, b). To confirm the silencing effects of M siSTAT3 or M siNEAT1 in vivo, we applied thermosensitive gel containing M siSTAT3-2 or M siNEAT1v2 to the mouse abdominal skin once per day for two days, then collected the skin samples for qPCR. The results showed that the RNA expression of STAT3 and NEAT1 was significantly inhibited by M siSTAT3-2 and M siNEAT1v2, respectively (Fig. 8c). We then generated an animal model with skin lesions caused by HSV-1 infection in C57BL/6 mice. The mice with skin lesions were treated with thermosensitive gels containing either control siRNA, M siSTAT3-2 or M siNEAT1v2. Interestingly, both M siSTAT3-2 and M siNEAT1v2 inhibited the development of the skin lesions and cured them quickly (Fig. 8d, e). These results suggest that NEAT1 and STAT3 are potential therapeutic targets for limiting viral replication.

## Discussion

Katsutoshi Imamura et al. previously reported that HSV-1 infection increases the expression of NEAT1 and the formation of paraspeckles [13]; however, the mechanism and significance remained unclear. In this study, we demonstrated that HSV-1 infection increases the expression of NEAT1 and the formation of paraspeckles in a STAT3-dependent manner. STAT3 regulates the expression of the *NEAT1* gene by binding to its promoter and enhancing its transcription. However, HSV-1 infection does not directly affect the expression of other paraspeckle components, such as P54nrb and PSPC1. Therefore, the effect of viral infection on the formation of paraspeckles is probably attributable to the increase in NEAT1 expression, which facilitates the organization of the paraspeckle components.

Although changes in NEAT1 expression have been implicated in several different viral infections [13, 20, 24], few studies have focused on the potential roles of NEAT1 in viral replication and viral gene expression. Here, we report that NEAT1 modulates HSV-1 replication. As the scaffolding RNA of paraspeckles, NEAT1 is known to exert its functions mainly through the nuclear retention of cellular RNAs that contain inverted Alu repeat elements in their 3′ untranslated regions (UTRs) [25]. Of the genes in the human genome, 333 are estimated to be potentially retained in paraspeckles [26]. However, the likelihood of the NEAT1-dependent nuclear retention of the ICP0 and TK mRNAs is very low because the 3′ UTRs of these viral transcripts are short (Fig. S7a), and they are unable to form the structure required for A-to-I editing [27].

Like other nuclear bodies [28, 29], the paraspeckles consist of nucleic acid and protein components. Specifically, paraspeckles contain NEAT1 and numerous RNA-binding proteins, such as P54nrb and PSPC1, which are involved in the formation of an extensively intertwined dimer through their coiled-coil domains. This dimer structure is critical for paraspeckle formation [30] and localizes within both the nucleoplasm and the paraspeckles [31]. Although several functions of the paraspeckle components have been previously reported, few studies have focused on the potential functions of paraspeckles as nuclear bodies. We show here that HSV-1 genes bind to NEAT1, P54nrb, and PSPC1, and co-localize in paraspeckles. Damaging the paraspeckle structure via silencing NEAT1, P54nrb, or PSPC1 significantly affects viral gene expression. These observations suggest that paraspeckles are functional nuclear sub-nuclear structures that are involved in HSV-1 replication.

STAT3 was originally recognized as a transcription factor that is activated by interleukin 6, and it has been shown to participate in multiple biological processes [32]. In response to stressful conditions, STAT3 is phosphorylated at tyrosine 705 by JAK2/TYK2 kinase, causing the dimerization of STAT3 and its translocation to the nucleus. A recent study showed that cytoplasmic lncRNA can bind directly to STAT3 to regulate its phosphorylation status [33], suggesting that STAT3 can bind to both RNA and DNA. Interestingly, we demonstrated that phosphorylated STAT3 Y705 binds to the promoters of ICP0 and TK and regulates their transcription after HSV-1 infection. This process is paraspeckle dependent. Through binding with STAT3, PSPC1 is required for the recruitment of STAT3 to paraspeckles and facilitates the interaction between STAT3 and viral genes, finally increasing viral gene expression. Knockdown of paraspeckle components affects this protein–DNA association rather than the expression level of STAT3 or the phosphorylation status of STAT3 (Fig. S7b, c). SFPQ, another essential paraspeckle protein, was reported to modulate interleukin-8 (IL-8) expression in HSV-1-infected cells [13]. In this study, we found that SFPQ negatively regulated viral gene expression by inhibiting the transcription of viral genes. In contrast to P54nrb and PSPC1, SFPQ functions as a competitive inhibitor of STAT3 in STAT3-mediated HSV-1 gene expression, suggesting that SFPQ may play a vital role in the host antiviral response.

In this article, we investigated the role of NEAT1 and paraspeckles in HSV-1 replication and viral gene expression. We found that HSV-1 infection upregulates NEAT1 expression and paraspeckle formation in a STAT3-dependent manner. Then, through binding with NEAT1 and other paraspeckle components, the viral genes were retained in paraspeckles. PSPC1, the paraspeckle component, was required for the recruitment of STAT3 to paraspeckles and facilitated the interaction between the viral genes and STAT3, finally increasing viral gene expression and viral replication (Fig. 8f). Overall, HSV-1 can use NEAT1, an lncRNA in host cells, and paraspeckles, sub-nuclear structures, to facilitate viral gene expression and enhance viral replication.

## Materials and methods

### Cell culture, HSV-1 infection, viral DNA extraction, and plaque assay

HeLa cells (American Type Culture Collection, ATCC) and mouse embryonic fibroblast (MEF) cells (ATCC) were grown in Dulbecco’s modified Eagle’s medium (Gibco/Invitrogen Ltd, 12800-017) containing 10% fetal bovine serum (PAA, A15-101) and 10 U/ml penicillin–streptomycin (Gibco/Invitrogen Ltd, 15140-122) in a humidified 5% CO_2_ incubator at 37 °C. The HeLa and MEF cells were infected with HSV-1 strain SM44 at an MOI of 1. To study the effects of NEAT1 on viral DNA replication in HSV-1-infected cells, viral DNA was extracted from the cell lysates with the Tissue/Cell Genomic DNA Extraction Kit (ChinaTopBio, DE1402-50) according to the manufacturer’s protocol. For the plaque-forming assay, Vero cells (Chinese Academy of Sciences) were inoculated with 100 μl of serially diluted viral fluid for 1 h. After viral adsorption, the HSV-1-infected cells were overlaid with medium containing 1% human serum for 48 h and stained with 1% crystal violet in 10% formaldehyde for 15 min to visualize the plaques.

### Plasmid construction

To generate the luciferase reporters for the promoter assay, we cloned the sequences upstream of *NEAT1* (Fragment 1, between −1798 and −1098 bp; Fragment 2, between −1104 and −65 bp) and the ICP0 promoter into pGL3-enhancer reporter at the *Kpn*I/*Xho*I sites. To confirm that STAT3 interacts with the NEAT1 promoter, two luciferase reporter constructs that inserted the sequences upstream from NEAT1 between −1561 and −1251 bp containing the STAT3-binding motif (NEAT1 WT) and with a deletion in the STAT3-binding motif (NEAT1 MUT) were purchased from Shanghai GenePharma Co. Ltd. We also used the pRL-TK reporter vector (Promega, E2241) for the luciferase assay because it contains the sequence of the HSV-1 TK gene promoter. All the constructs were confirmed with DNA sequencing. The primers used are listed in Table S1.

### Cell transfection, RNA isolation, reverse transcription, and qPCR

All of the synthetic siRNAs and the negative control (siCTRL) siRNA were purchased from Shanghai GenePharma Co., Ltd. All the siRNAs were transfected with Lipofectamine™ 2000 transfection reagent (Invitrogen, 11668-019) according to the manufacturer’s protocol. The sequences of the siRNAs used are listed in Table S1. For plasmid transfection, the cells were transiently treated with Lipofectamine™ 3000 (Invitrogen, 1656200). Total RNA was isolated with RNAiso Plus (Takara, D9108B) according to the manufacturer’s protocol. Real-time PCR was performed with ReverTra Ace^®^ qPCR RT Master Mix with gDNA remover (Toyobo, FSQ-301) and SYBR Green PCR Master Mix (Toyobo, QPK-201). All mRNA levels were measured and normalized to that of β-actin mRNA. The primers used are listed in Table S1.

### Western blotting

Cells were lysed in ice-cold whole cell extract buffer B [50 mM Tris–HCl (pH 8.0), 4 M urea, and 1% Triton X-100] supplemented with complete protease inhibitor cocktail (Roche). The cell extracts were resolved with SDS-PAGE and analyzed with western blotting. The protein bands were visualized with ECL Blotting Detection Reagents. The antibodies used for western blotting included an anti-P54nrb antibody (Abcam, ab70335), anti-PSPC1 antibody (Abcam, ab104238), anti-SFPQ antibody (Abcam, ab11825), anti-STAT3 antibody (CST, 124H6), anti-pSTAT3Y705 antibody (Abcam, ab76315), anti-HSV-1 ICP0 antibody (Abcam, ab6513), anti-HSV-1 TK antibody (Santa Cruz Biotechnology, sc-28037), and anti-β-actin antibody (Proteintech, 60008-1-Ig).

### Luciferase assay

For luciferase reporter assays, 100 ng luciferase reporter plasmids and 20 pmol siRNA were co-transfected into 10^4^ HeLa cells in a 24-well culture plate; each experiment was repeated in triplicate. Transfected cells were lysed 24 h after transfection, and luciferase activities were assayed with a Dual-Luciferase Reporter System following the manufacturer’s instructions (Promega, E1960) and normalized to the total protein content of the cell lysate. The experiment was carried out in triplicate.

### ChIP assay

The ChIP assay was performed with Dahl’s protocol [34]. In brief, cells were fixed with 1% formaldehyde and sonicated to shear the DNA to an average fragment size of 200–1000 bp. After centrifugation, the supernatants were incubated with the indicated primary antibodies. Chromatin DNA was purified with Dynabeads Protein G (Invitrogen, 10004D) and subjected to real-time PCR. The region-specific primers used are listed in Table S1.

### RNA/DNA FISH and immunofluorescence microscopy

To detect human NEAT1, RNA-FISH was performed with a NEAT1-specific probe (Biosearch Technologies, SMF-2037-1) as described in [35]. The NEAT1 probe consists of a set of Quasar^®^ 570-labeled oligos and is designed against the middle segment (3800–11,700 bp) of human NEAT1v2. To confirm the effects of NEAT1 and the paraspeckle protein components on HSV-1 replication, HeLa cells were transfected with NEAT1 siRNA, P54nrb siRNA, or PSPC1 siRNA for 36 h. Twelve hours after HSV-1 infection, the cells were fixed in 4% paraformaldehyde for 10 min. The cells were blocked and incubated with an anti-HSV-1 glycoprotein antibody (Abcam, ab9533) for 1 h and then with a secondary antibody for 1 h. The cells were then washed, counterstained with DAPI, and mounted for observation. To determine whether NEAT1 co-localized with the HSV-1 genomic DNA, RNA/DNA co-FISH was performed as described in [36]. Briefly, HeLa cells infected with HSV-1 were incubated with the NEAT1 probe overnight at 37 °C and then incubated with biotin-labeled partial HSV-1 DNA at 42 °C for 12–16 h. After blocking, the cells were stained with an anti-biotin antibody (ImmunoReagents, GtxOt-070-D488NHSX) for 1 h. The cells were then washed, counterstained with DAPI, and mounted for observation. To study the interaction between P54nrb, PSPC1, and HSV-1 genomic DNA, HeLa cells infected with HSV-1 were incubated with biotin-labeled HSV-1 genomic DNA overnight at 42 °C. To detect co-localization, the cells were fixed in 4% formaldehyde for 5 min and incubated with an anti-P54nrb antibody (Abcam, ab70335) or an anti-PSPC1 antibody (Abcam, ab104238) for 1 h. After the cells were washed and incubated with the secondary antibody, they were counterstained with DAPI and mounted for observation. To study the interaction between PSPC1 and pSTAT3 Y705, HeLa cells infected with HSV-1 were incubated with an anti-PSPC1 antibody (Abcam, ab104238) and an anti-pSTAT3 Y705 antibody (Santa Cruz Biotechnology, sc-7993) for 1.5 h at room temperature. After the cells were washed and incubated with the secondary antibody, they were counterstained with DAPI and mounted for observation. To study the role of PSPC1 in the interaction between NEAT1 and pSTAT3 Y705, HeLa cells were transfected with PSPC1 siRNA or negative control siRNA for 36 h. Four hours after HSV-1 infection, the cells were incubated with the NEAT1 probe overnight at 37 °C and then incubated with the anti-pSTAT3 Y705 antibody (Santa Cruz Biotechnology, sc-7993) for 1.5 h at room temperature. After the cells were washed and incubated with the secondary antibody, they were counterstained with DAPI and mounted for observation. Cell images were obtained with an Olympus FV1000 confocal microscope.

### Zosteriform model of infection

The zosteriform model of infection was established as described in [37]. Briefly, the flank of each C57BL/6 mouse was clipped and depilated with Nair™ Sensitive Hair Removal Cream (Reckitt Benckiser), and a 20-μl droplet containing 10^6^ PFU of virus was applied to the flank over the spleen. The skin was scarified 20 times with a 27-gauge needle through the droplet. The mice were then anesthetized with ether for an additional 10 min to allow the viral suspension to dry. When the infected mice developed a zosteriform lesion at the inoculation site, the complex of 2-*O*-methyl modified M siSTAT3-2, M siNEAT1v2 or the negative control siRNA and transferrin-polyethylenimine (TF-PEI) was placed on the mouse skin with a thermosensitive gel once per day for two days. The width of the zosteriform lesion was used as an index of the severity of the lesion. All experiments were performed with 6- to 8-week-old mice with the approval of the Animal Welfare and Ethics Committee of Tsinghua University.

### Statistical analysis

Each experiment was repeated three times. The results are presented as the mean ± SD; *p < 0.01, **p < 0.05. Comparisons between two groups were evaluated with a two-sample *t* test. For three or more groups, standard one-way analysis of variance (ANOVA) followed by Bonferroni’s test for multiple comparisons was completed. A two-tailed probability value <0.05 was considered statistically significant.

### Electronic supplementary material

Below is the link to the electronic supplementary material.

Supplementary material 1 (DOCX 2845 kb)
