# Supplementary material for: NEAT1 modulates herpes simplex virus-1 replication by regulating viral gene transcription
Source: Cell Mol Life Sci. 2016 Oct 25;74(6):1117–31. doi: 10.1007/s00018-016-2398-4 (PMC5309293; doi:10.1007/s00018-016-2398-4)
Supplement: Supplementary file 1 — Supplementary material 1 (DOCX 2845 kb) [file 18_2016_2398_MOESM1_ESM.docx]

**NEAT1 Modulates Herpes Simplex Virus**-**1 Replication by Regulating Viral Genes Transcription**

**Ziqiang Wang^1,2†^**, **Ping Fan^1,2†^**, **Yiwan Zhao^1,2^**, **Shikuan Zhang^2^**, **Jinhua Lu^3^**, **Weidong Xie^2^**, **Yuyang Jiang^4^**, **Fan Lei^5^**, **Naihan Xu^2, 6^***, and **Yaou Zhang^2, 6^***

^1^School of Life Sciences, Tsinghua University, Beijing 100084, P.R. China

^2^Key Lab in Healthy Science and Technology, Division of Life Science, Graduate School at Shenzhen, Tsinghua University 518055, Shenzhen, P.R. China

^3^Shenzhen South China Pharmaceutical Co., Ltd, Shenzhen 518055, P.R. China

^4^The State Key Laboratory Breeding Base-Shenzhen Key Laboratory of Chemical Biology, the Graduate School at Shenzhen, Tsinghua University, Shenzhen 518055, P. R. China

^5^School of Pharmaceutical Sciences, Tsinghua University, Beijing 100084, P.R. China

^6^Open FIESTA Center，Tsinghua University, Shenzhen 518055, P.R. China

†These authors contributed equally to this paper.

*To whom correspondence should be addressed to Yaou Zhang. Email: zhangyo@sz.tsinghua.edu.cn, Correspondence may also be addressed to Naihang Xu. Email: xu.naihan@sz.tsinghua.edu.cn

*Corresponding author, Key Lab in Healthy Science and Technology, Division of Life Science, Graduate School at Shenzhen, Tsinghua University, Shenzhen, P.R. China, phone: (86) 755-26036884, fax: (86) 755-26036884

E-mail: zhangyo@sz.tsinghua.edu.cn

**Supplementary material**

**
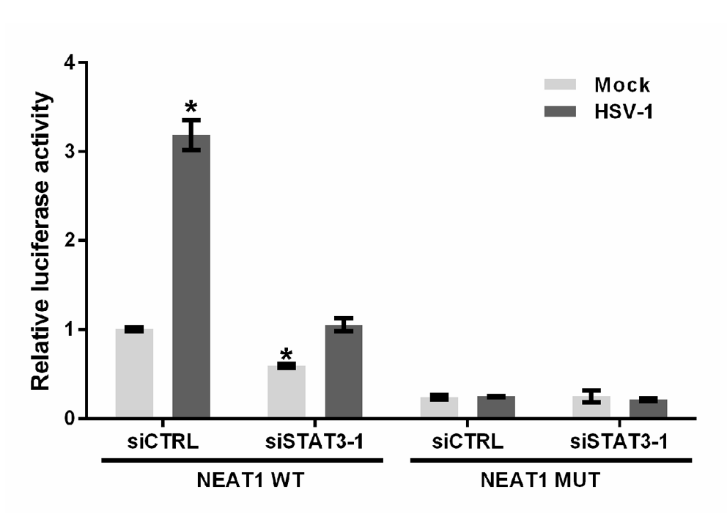
**

**Fig. S1 STAT3 promotes the transcription of *NEAT1* in response to HSV-1 infection.** Luciferase activity assay in HeLa cells transfected with STAT3 siRNA and the NEAT1 WT or NEAT1 MUT construct. The data were normalized to the control level in mock-infected cells. *p < 0.01.

**
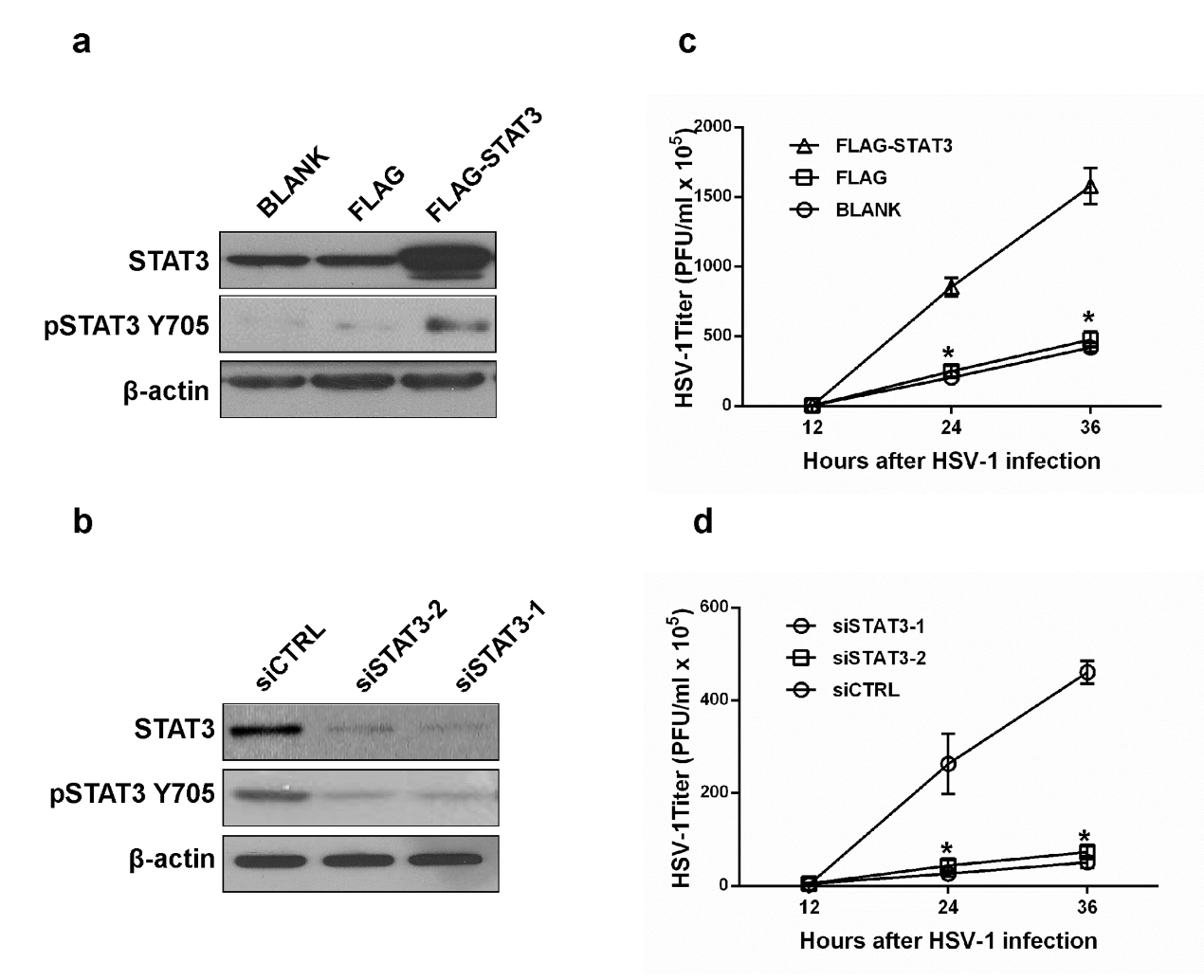
**

**Fig. S2 STAT3 modulates HSV-1 replication in HeLa cells.** HeLa cells were transfected with FLAG-STAT3 (**a**) or siSTAT3 (**b**) for 36 h. The levels of STAT3 and pSTAT3 Y705 were determined with western blotting. Plaque-forming assay in HeLa cells transfected with FLAG-STAT3 (**c**) or siSTAT3 (**d**) and infected with HSV-1 at the indicated time points. *p < 0.01.


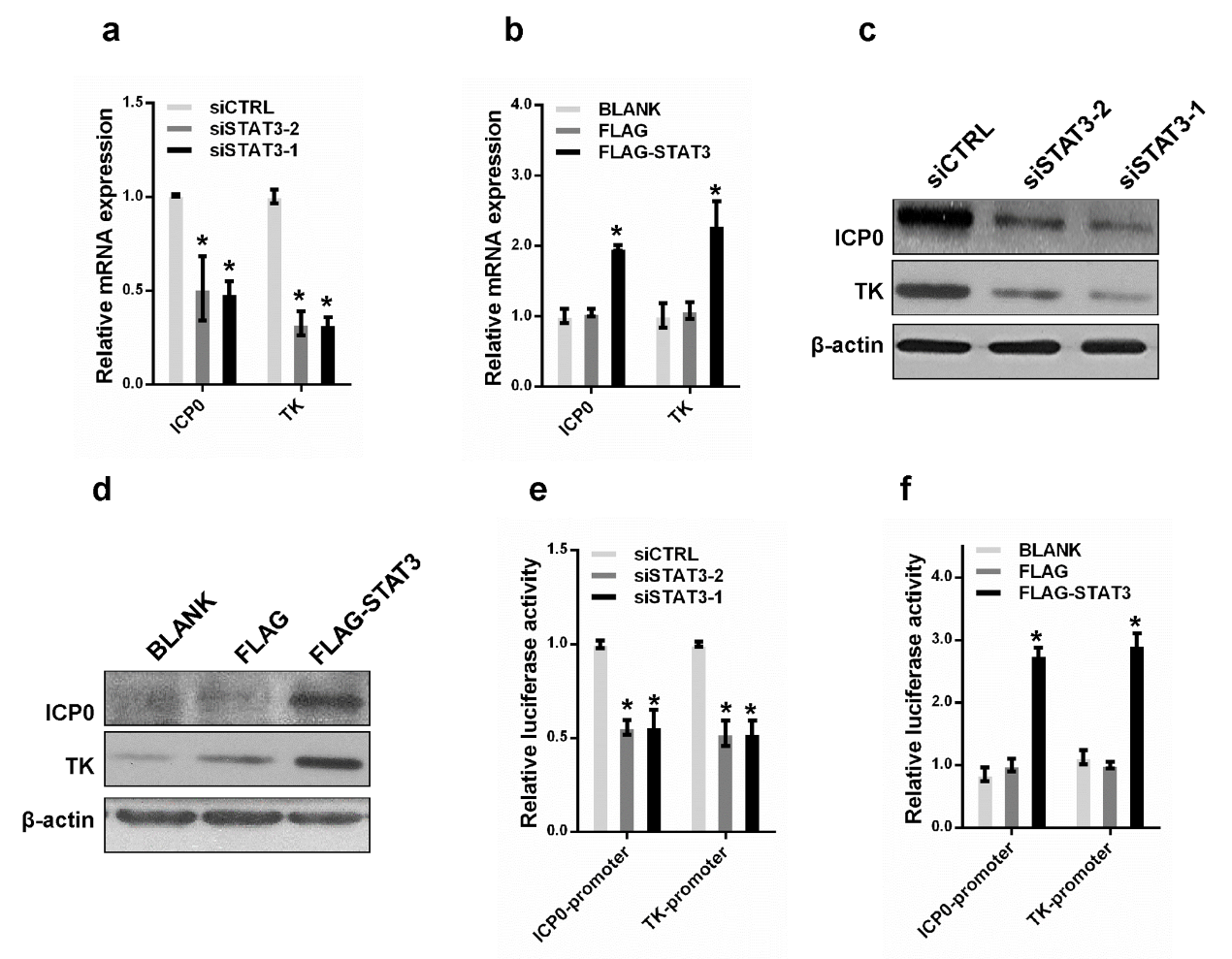


**Fig. S3 STAT3 modulates HSV-1 gene expression by affecting promoter activity.** The mRNA expression levels of ICP0 and TK relative to that of *ACTB* were determined in HeLa cells transfected with STAT3 siRNA (**a**) or FLAG-STAT3 (**b**) and infected with HSV-1 for 4 h with real-time PCR. Western blot analysis of ICP0, TK and β-actin in HeLa cells transfected with STAT3 siRNA (**c**) or FLAG-STAT3 (**d**) and infected with HSV-1 for 4 h. Luciferase activity assay in HeLa cells co-transfected with STAT3 siRNA (**e**) or FLAG-STAT3 (**f**) and a luciferase reporter plasmid containing either the ICP0 promoter or the TK promoter. *p < 0.01.


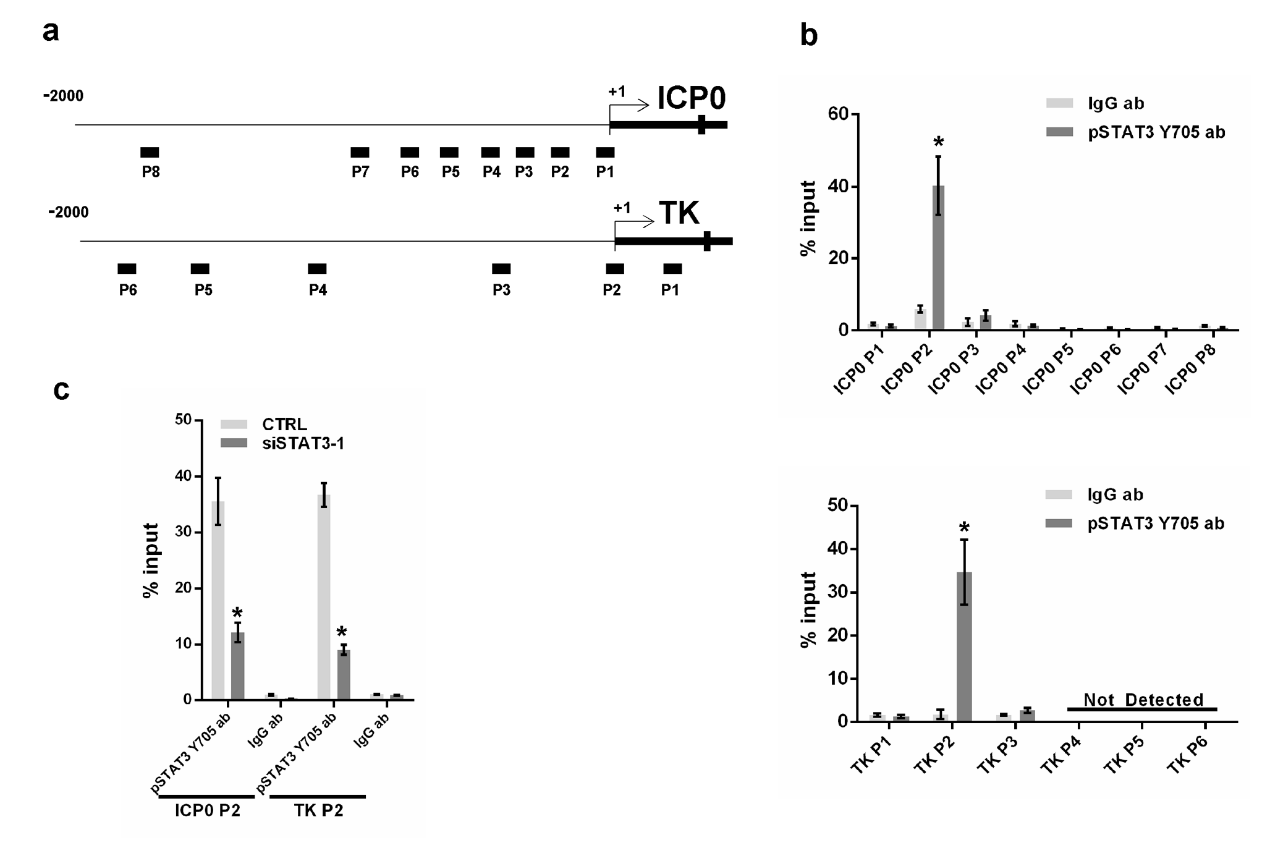


**Fig. S4 STAT3 associates with HSV-1 viral gene promoters. a** Schematic diagram showing the gene structure of ICP0 and TK, in which the black boxes represent the primer-amplified regions. **b** HeLa cells were infected with HSV-1 for 4 h. ChIP assays were performed with an anti-pSTAT3 Y705 antibody or an anti-IgG antibody. The fold enrichment of the ICP0 and TK promoters by pSTAT3 Y705 relative to the input level was examined with real-time PCR. **c** After transfection for 36 h with STAT3 siRNA or the negative control, HeLa cells were infected with HSV-1 for 4 h. ChIP assays were performed with an anti-pSTAT3 Y705 antibody or an anti-IgG antibody, and the fold enrichment of the ICP0 P2 region and the TK P2 region by pSTAT3 Y705 relative to the input level was examined with real-time PCR. *p < 0.01.


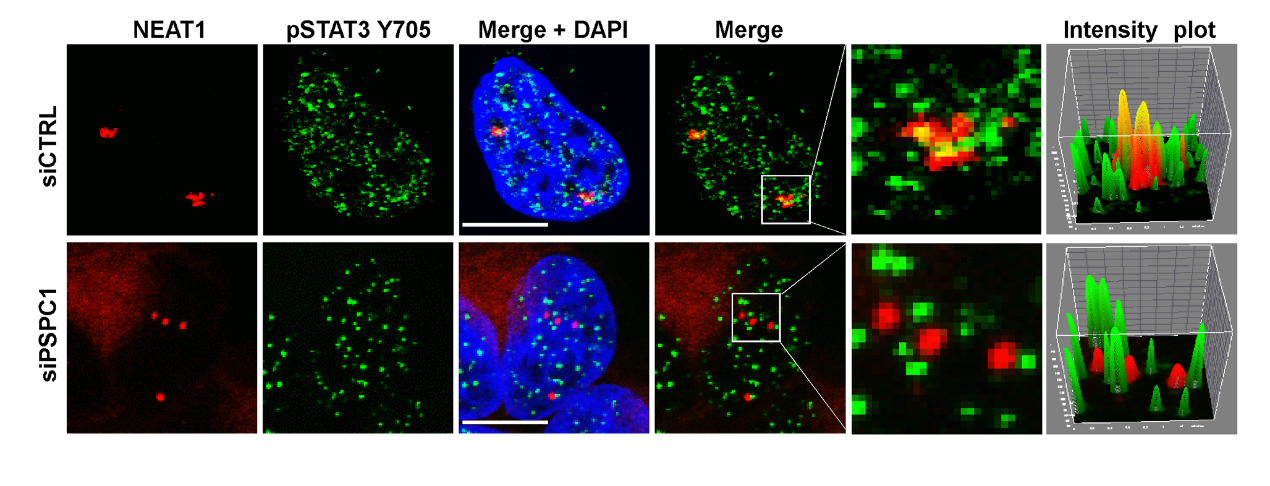


**Fig. S5** **PSPC1 recruits STAT3 to paraspeckles.** HeLa cells transfected with PSPC1 siRNA (siPSPC1) or negative control siRNA (siCTRL) were infected with HSV-1 for 4 hours. The cells were incubated with NEAT1 (red) and then with pSTAT3 Y705 (green) before being subjected to confocal analysis. The intensity plots for the red and green channels were analyzed with ImageJ software. Scale bars, 10 μm.


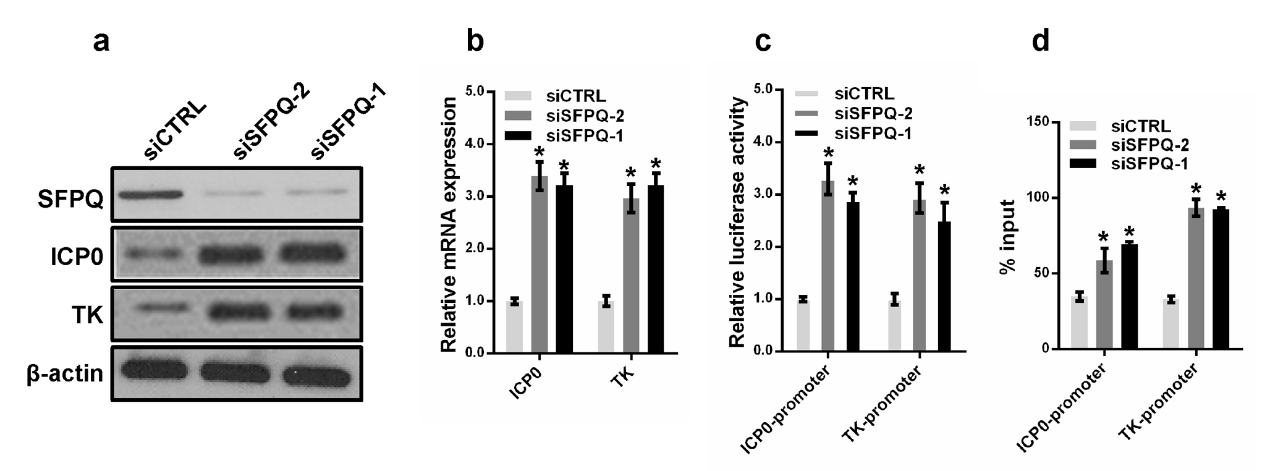


**Fig. S6** **SFPQ is involved in STAT3-mediated viral gene expression.** **a** Western blot analysis of SFPQ, ICP0, TK and β-actin in HeLa cells transfected with SFPQ siRNA or negative control siRNA and infected with HSV-1. **b** HeLa cells transfected with the indicated siRNAs were infected with HSV-1 for 4 h. The expression of ICP0 and TK relative to that of β-actin was measured with real-time PCR. **c** Luciferase activity assay in HeLa cells co-transfected with SFPQ siRNA and a luciferase reporter plasmid containing either the ICP0 promoter or the TK promoter. **d** HeLa cells transfected with SFPQ siRNA or negative control siRNA were subjected to HSV-1 infection. Samples were collected for ChIP assays to analyze the fold enrichment of the ICP0 and TK promoters by pSTAT3 Y705 relative to the input level. *p < 0.01.


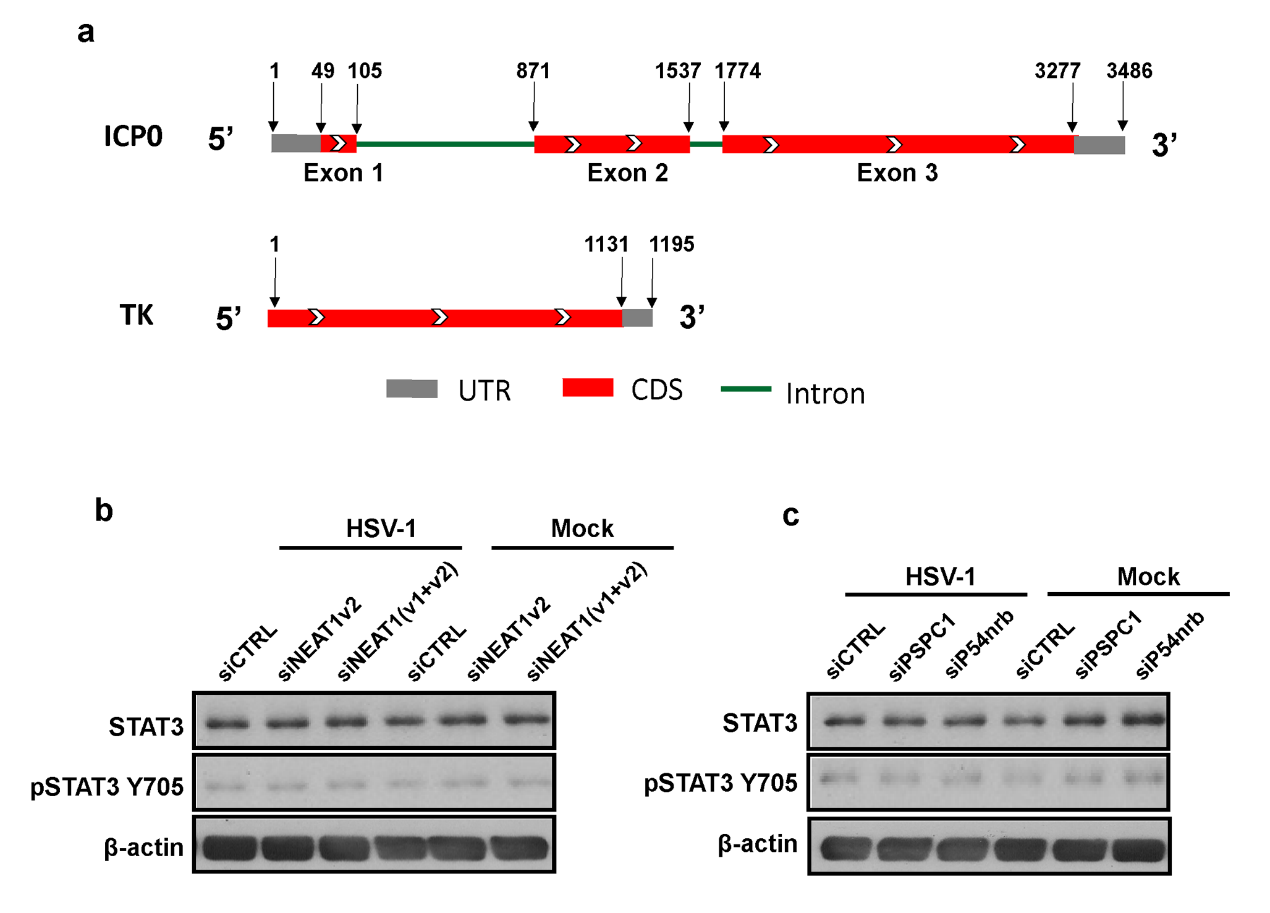


**Fig. S7 a** Schematic diagram showing the RNA structures of ICP0 and TK. Western blot analysis of STAT3 and pSTAT3 Y705 in HeLa cells transfected with NEAT1 siRNA (**b**), P54nrb siRNA or PSPC1 siRNA (**c**) and mock infected or infected with HSV-1 for 4 h.

**Table S1. Sequences of primers and siRNAs used in this study**

**Name sense sequence antisense sequence**

| \| **SiRNA** \| \| \|  \| \| --- \| --- \| --- \| --- \| \| hNEAT(v1+v2) siRNA \| GUGAGAAGUUGCUUAGAAA \| UUUCUAAGCAACUUCUCAC \|  \| \| hNEAT1v2 siRNA \| CAAACUCUGUACCCAUUAA \| UUAAUGGGUACAGAGUUUG \|  \| \| mNEAT(v1+v2) siRNA \| CCAGCAGCUUUCAAGACCA \| UGGUCUUGAAAGCUGCUGG \|  \| \| mNEAT1v2 siRNA \| ACAGCAUACUGGCCAGAAA \| UUUCUGGCCAGUAUGCUGU \|  \| \| hP54nrb siRNA \| CCAGCAAUUUCACAAGGAA \| UUCCUUGUGAAAUUGCUGG \|  \| \| mP54nrb siRNA \| CCAGGAGAGAAGACCUUUA \| UAAAGGUCUUCUCUCCUGG \|  \| \| hPSPC1 siRNA \| GAACCUUUCUCCAGUUGUU \| AACAACUGGAGAAAGGUUC \|  \| \| mPSPC1 siRNA \| GCAAGUACGUAUUGAGAAA \| UUUCUCAAUACGUACUUGC \|  \| \| hSFPQ siRNA-1 \| GAACUUCACAAUCAAGAAA \| UUUCUUGAUUGUGAAGUUC \|  \| \| hSFPQ siRNA-2 \| GCCAAUUUGUCUCUCUUGA \| UCAAGAGAGACAAAUUGGC \|  \| \| hSTAT3-1 siRNA \| GAAGGAGGCGUCACUUUCA \| UGAAAGUGACGCCUCCUUC \|  \| \| hSTAT3-2 siRNA \| GGAAAUGUCUUGUGUUGUU \| AACAACACAAGACAUUUCC \|  \| \| mSTAT3-1 siRNA \| CCUUGCUAAUAUCCACAUA \| UAUGUGGAUAUUAGCAAGG \|  \| \| mSTAT3-2 siRNA \| AUCAUCAUGGGCUAUAAGA \| UCUUAUAGCCCAUGAUGAU \|  \| \| Negative control siRNA \| UUCUCCGAACGUGUCACGU \| ACGUGACACGUUCGGAGAA \|  \| \| **Primers pairs for Real Time PCR** \| \| \| \| \| hNEAT1(v1+v2) \| GAGAACCAAAGGGAGGGGTG \| TGCTGCGTATGCAAGTCTGA \|  \| \| hNEAT1V2 \| ACATTGTACACAGCGAGGCA \| CATTTGCCTTTGGGGTCAGC \|  \| \| mNEAT1(v1+v2) \| GGTTCCAGGCACAATCCTCA \| ACCTCATGAGTGCTTTGCCA \|  \| \| mNEAT1v2 \| CTTGCCACACCTTGTCTTGC \| TAGCTGGTGCATCCTGTGTG \|  \| \| ICP0 \| CCCACTATCAGGTACACCAGCTT \| CTGCGCTGCGACACCTT \|  \| \| TK \| CGATGACTTACTGGCGGGTGT \| GCGTCGGTCACGGCATAA \|  \| \| mSTAT3 \| TGTGACACCATTCATTGATGCAG \| GATATGGGGTTCGGCTGCTTA \|  \| \| hActin-beta \| TGACGTGGACATCCGCAAAG \| CTGGAAGGTGGACAGCGAGG \|  \| \| mActin-beta \| GTACCCAGGCATTGCTGACA \| CGCAGCTCAGTAACAGTCCG \|  \| \| **Primer pairs for plasmid constructions** \| \| \|  \| \| NEAT1 Fragment 1 \| CGGGGTACCGGGCCCAGAAACAGCACTAC \| CCGCTCGAGAGGACTTTGGACCGTGTAGC \|  \| \| NEAT1 Fragment 2 \| CGGGGTACCAAGTCCTCTCCAGACATTCGG \| CCGCTCGAGGGGACGATTCCTCCACGG \|  \| \| ICP0-promoter \| CGGGGTACCTCTAACGTTACACCCGAGGC \| CCGCTCGAGTTCTGTGGTGATGCGGAGAG \|  \| \| hSTAT3 \| CCCAAGCTTATGGCCCAATGGAATCAGCTA \| CCGCTCGAGCATGGGGGAGGTAGCGCA \|  \| |  |  |
| --- | --- | --- | --- | --- | --- | --- | --- | --- | --- | --- | --- | --- | --- | --- | --- | --- | --- | --- | --- | --- | --- | --- | --- | --- | --- | --- | --- | --- | --- | --- | --- | --- | --- | --- | --- | --- | --- | --- | --- | --- | --- | --- | --- | --- | --- | --- | --- | --- | --- | --- | --- | --- | --- | --- | --- | --- | --- | --- | --- | --- | --- | --- | --- | --- | --- | --- | --- | --- | --- | --- | --- | --- | --- | --- | --- | --- | --- | --- | --- | --- | --- | --- | --- | --- | --- | --- | --- | --- | --- | --- | --- | --- | --- | --- | --- | --- | --- | --- | --- | --- | --- | --- | --- | --- | --- | --- | --- | --- | --- | --- | --- | --- | --- | --- | --- | --- | --- | --- | --- | --- | --- | --- | --- | --- | --- | --- |
| \| **Primers pairs for ChIP** \| \| \| \| --- \| --- \| --- \| \| hNEAT1 P1 \| TGCCACATCACCACCTTCTG \| GAAGACATTTCGCCTGCGTC \| \| hNEAT1 P2 \| TGTCCCTCGGCTATGTCAGA \| GAGGGGACGTGTTTCCTGAG \| \| ICP0-promoter \| CCATTGGGGGAATCGTCAC \| CTTCTGTGGTGATGCGGAG \| \| TK-promoter \| AAACGCGGGCGTATTGGT \| ACAATACCGGAAGGAACCCG \| \| ICP0-P1 \| CCGACAGTCTGGTCGCATTT \| GGCTCCATGGGGGTCGTAT \| \| ICP0-P2 \| CCATTGGGGGAATCGTCAC \| CTTCTGTGGTGATGCGGAG \| \| ICP0-P3 \| GGGCATGCTAATGGGGTTCT \| GCAGTGACGATTCCCCCAAT \| \| ICP0-P4 \| CAATGAACCCGCATTGGTCC \| AGAACCCCATTAGCATGCCC \| \| ICP0-P5 \| CTTAATGGGCAACCCCGGTA \| CGCCTTCCCGAAGAAACTCA \| \| ICP0-P6 \| CTTGTTCCGCTTCCCGGTAT \| GAATACCGGGGTTGCCCATT \| \| ICP0-P7 \| TCTAACGTTACACCCGAGGC \| CGTATATATGCGCGGCTCCT \| \| ICP0-P8 \| AGACAGGCAAGCACTACTCG \| TGGAGGTTACCTGGGACTGT \| \| TK-P1 \| GGGCGATTGGTCGTAATCCA \| CCCAACGGCGACCTGTATAA \| \| TK-P2 \| AAACGCGGGCGTATTGGT \| ACAATACCGGAAGGAACCCG \| \| TK-P3 \| GCAGGTAGGTCTTCGGGATG \| GGCATCTCTGCCCCTTCTTC \| \| TK-P4 \| AAAAGCCTAGCAGGTCGGAG \| CCTCTCTTCTGGCGCCTAAC \| \| TK-P5 \| AGAAACTCGGCATACAGGGC \| GAGATTCTGGAGCGCGAACA \| \| TK-P6 \| AAGTGGTCCGGAAGCCAAAA \| GAGCCTTCTGATAGCCTCGG \| |  |  |
